# Stimulation of Transforming Growth Factor-β1-Induced Endothelial-To-Mesenchymal Transition and Tissue Fibrosis by Endothelin-1 (ET-1): A Novel Profibrotic Effect of ET-1

**DOI:** 10.1371/journal.pone.0161988

**Published:** 2016-09-01

**Authors:** Peter J. Wermuth, Zhaodong Li, Fabian A. Mendoza, Sergio A. Jimenez

**Affiliations:** 1 Jefferson Institute of Molecular Medicine, Thomas Jefferson University, Philadelphia, PA, United States of America; 2 Division of Rheumatology, Department of Medicine, Thomas Jefferson University, Philadelphia, PA, United States of America; Boston University Henry M Goldman School of Dental Medicine, UNITED STATES

## Abstract

TGF-β-induced endothelial-to-mesenchymal transition (EndoMT) is a newly recognized source of profibrotic activated myofibroblasts and has been suggested to play a role in the pathogenesis of various fibrotic processes. Endothelin-1 (ET-1) has been implicated in the development of tissue fibrosis but its participation in TGF-β-induced EndoMT has not been studied. Here we evaluated the role of ET-1 on TGF-β1-induced EndoMT in immunopurified CD31^+^/CD102^+^ murine lung microvascular endothelial cells. The expression levels of α-smooth muscle actin (α-SMA), of relevant profibrotic genes, and of various transcription factors involved in the EndoMT process were assessed employing quantitative RT-PCR, immunofluorescence histology and Western blot analysis. TGF-β1 caused potent induction of EndoMT whereas ET-1 alone had a minimal effect. However, ET-1 potentiated TGF-β1-induced EndoMT and TGF-β1-stimulated expression of mesenchymal cell specific and profibrotic genes and proteins. ET-1 also induced expression of the TGF-β receptor 1 and 2 genes, suggesting a plausible autocrine mechanism to potentiate TGF-β-mediated EndoMT and fibrosis. Stimulation of TGF-β1-induced skin and lung fibrosis by ET-1 was confirmed *in vivo* in an animal model of TGF-β1-induced tissue fibrosis. These results suggest a novel role for ET-1 in the establishment and progression of tissue fibrosis.

## Introduction

Activated myofibroblasts comprise a unique population of mesenchymal cells that play a crucial role in the development of pathologic fibrotic processes and are considered to be the ultimate effector cells in various fibrotic disorders including Systemic Sclerosis (SSc), Idiopathic Pulmonary Fibrosis (IPF), and cardiac, liver and kidney fibrosis [1,2]. These cells express high levels of α-smooth muscle actin (α-SMA) and display a remarkable pro-fibrotic phenotype with increased production of numerous extracellular matrix (ECM) macromolecules including type I and type III collagens. Owing to their crucial role in the pathogenesis of tissue fibrosis and various fibrotic diseases there has been intense investigation of their cellular origins [3,4]. These studies have shown that myofibroblasts arise from various sources including resident quiescent fibroblasts [4,5], bone marrow-derived fibrocytes [6–8] and epithelial cells undergoing epithelial to mesenchymal transition (EMT) [9–11]. More recently, it has also been demonstrated that endothelial cells are capable of acquiring a mesenchymal phenotype through endothelial to mesenchymal transition or EndoMT [12]. Although the occurrence of EndoMT has been well documented during vertebrate cardiac embryonic development [12], its post-developmental occurrence was not accepted until numerous recent studies demonstrated EndoMT in various experimental animal models of fibrosis and in various human fibrotic diseases [13–15]. Indeed, EndoMT has been shown to occur in organ-specific fibrotic disorders including cardiac, kidney, and intestinal fibrosis [16–21], in cancer-associated fibrosis [22], and in Systemic Sclerosis (SSc)-associated pulmonary fibrosis and Pulmonary Arterial Hypertension (PAH) as well as in Primary PAH [23–26].

TGF-β is a member of a large family of multifunctional polypeptide growth factors involved in the regulation of a broad array of biological and physiological processes [27,28]. TGF-β signaling results in a potent stimulation of expression of a large number of pro-fibrotic genes and in a marked increase in the production of their corresponding proteins [27–29] and has been shown to play a major role in the development of tissue fibrosis in numerous organ-specific and systemic human fibrotic diseases including SSc [30–32]. Recent studies have shown that besides its effects on the expression of pro-fibrotic genes TGF-β pro-fibrotic effects may also be mediated through its key role in the initiation and regulation of EndoMT [13–15, 17, 33–35].

Endothelin-1 (ET-1), is a potent vasoconstrictor polypeptide produced and secreted by endothelial cells [36,37]. ET-1 plays a crucial role in the pathophysiology of PAH and is a prime therapeutic target for PAH and related group of disorders [38,39]. Besides its vascular effects, numerous studies have described a variety of ET-1 profibrogenic activities including stimulation of the synthesis of types I and III collagens, inhibition of the production of matrix degrading metalloproteinases, stimulation of EMT, and induction of expression of profibrogenic cytokines and growth factors such as connective tissue growth factor [40–46]. Furthermore, various human fibrotic diseases have been shown to display increased production of ET-1 [47–49]. However, the role of ET-1 in EndoMT induction or in TGF-β-induced EndoMT has not been studied extensively. One study [50] showed that endothelial cell-derived ET-1 promotes cardiac fibrosis and heart failure in diabetic hearts through stimulation of EndoMT, and a more recent study employing immunopurified CD31^+^ dermal endothelial cells from SSc patients showed that TGF-β and ET-1 induced EndoMT in normal and SSc endothelial cells, that these effects involved the Smad pathway, and that they were blocked by the specific ET-1 receptor antagonist, macitentan [26].

The purpose of the studies described here was to examine the interactions between ET-1 and TGF-β1 in the induction of EndoMT in murine lung microvascular endothelial cells and to identify changes in expression levels of various relevant genes participating in EndoMT modulation. These studies showed that ET-1 caused a strong potentiation of TGF-β1-induced EndoMT *in vitro*. We further present *in vivo* results confirming the potentiation of TGF-β1-induced tissue fibrosis in a murine animal model of fibrosis. Collectively, these results provide strong experimental evidence supporting a novel mechanism for the profibrotic effects of ET-1 and suggest that inhibition of ET-1 activity during the earliest stages of development of fibrotic processes when EndoMT plays an important role may represent a potent and effective antifibrotic approach.

## Methods

### Materials

Complete endothelial cell culture medium consisting of basal ECM supplemented with 5% FBS, 10% endothelial cell growth supplement, 100 U/mL penicillin and 100 μg/mL streptomycin was purchased from ScienCell Research Laboratories (Carlsbad, CA). Anti-CD31 and anti-CD102 antibodies were purchased from BD Biosciences (Bedford, MA) and fetal bovine serum (FBS) from Atlanta Biological (Lawrenceville, GA). The anti-α-SMA and anti-GAPDH antibodies were from Millipore (Billerica, MA). TGF-β1 was from Peprotech (Rocky Hill, NJ), and ET-1 was from R&D Systems (Minneapolis, MN). Bosentan was purchased from Selleck Chemicals (Houston, TX) and was resuspended in DMSO before use.

### Isolation of Murine Pulmonary Endothelial Cells

Pulmonary microvascular endothelial cells were isolated from 8–12 week-old C57BL6/J mice (Jackson Laboratories, Bar Harbor, ME; generation F25) with a modification of the method of Marelli-Berg et al. [51] as described previously [52,53]. The procedures employed for isolation of murine endothelial cells were approved by the Thomas Jefferson University Institutional Animal Care and Use Committee and performed in accordance with National Institutes of Health guidelines. Briefly, murine lungs were harvested, minced, and enzymatically digested with collagenase (30 mg/100ml in 0.1% BSA, Worthington, Lakewood, NJ) to obtain a single cell suspension. Endothelial cells were immunoselected with rat anti-mouse CD31 antibody followed by magnetic bead separation using goat anti-rat IgG-conjugated microbeads (1:5, Miltenyi Biotec, Auburn, CA, USA). The isolated endothelial cells were cultured in complete endothelial cell culture medium on 2% gelatin pre-coated tissue culture dishes for 3–5 days. Following expansion, the cells were resuspended and a second immunologic separation was carried out using rat anti-mouse CD102 antibody as described [52,53]. The resulting CD31^+^/CD102^+^ cells were plated and the endothelial cell phenotype of the preparation was confirmed by evaluating cellular uptake of 1,1'-dioctadecyl-3,3,3',3'-tetramethylindocarbocyanine perchlorate (DiI)-acetylated LDL (DiI-AcLDL, Biomedical Technologies, Stoughton, MA) and assessment of cell morphology as described [52,53]. The proportion of spindle-shaped mesenchymal cells relative to polygonal-shaped endothelial cells was assessed employing the Cell Counter.jar plugin for NIH ImageJ software as described [54]. Cells with a diameter at their longest axis that was two-fold greater than the average diameter of untreated cobblestone endothelial cells were considered spindle-shaped.

### Culture and Treatment of Murine Lung Microvascular Endothelial, Cells with TGF-β1, ET-1, and Bosentan

The immunopurified murine lung microvascular endothelial cells were washed with serum-free medium and then incubated with either TGF-β1 (10 ng/mL), or ET-1 (100 ng/mL), or TGF-β1 plus ET-1 in FBS-depleted (0.5% FBS) endothelial cell culture medium. To confirm the effects of ET-1, parallel samples were also pretreated with the dual ET-1 receptor antagonist, Bosentan (10 μM) for 3 h, after which the medium was replaced with fresh endothelial cell culture medium containing 0.5% FBS and either ET-1 plus Bosentan or TGF-β1 plus ET-1 plus Bosentan. The media were changed at 24 h with media containing fresh additives. On day 3 the cells were harvested and total RNA and cellular proteins were isolated for subsequent studies. Cytotoxicity and effects on cellular proliferation were assessed by incubating cells plated at equal density prior to the initiation of treatment with the tetrazolium salt WST-1 (Roche Diagnostics, Indianapolis, IN) for 2 h at 37°C in a CO_2_ incubator and measuring the absorbance at a wavelength of 450 nm. Differences in absorbance levels reflect increases or decreases in cell numbers mediated by changes in cellular proliferation and/or cytotoxicity effects.

### Immunofluorescence Staining

Murine lung microvascular endothelial cells were seeded onto glass culture slides and treated as described above. Following treatment, the cells were fixed with 3.7% formaldehyde and permeabilized with 0.1% Triton X-100 in PBS for 3 min. Slides were washed with PBS and blocked with PBS containing 1% BSA at room temperature for 1 h, and then they were incubated with primary antibodies against α-SMA (1:200). Slides were then incubated with Cy3-conjugated secondary antibodies (1:500) followed by 4,6-diamidino-2-phenylindole (DAPI) (Jackson ImmunoResearch Laboratories, West Grove, PA) for nuclear staining.

### Western Blot Analysis

The cells were washed with cold PBS, harvested with a cell lifter, centrifuged and lysed with RIPA lysis buffer (25 mM Tris-HCl pH 7.6, 150 mM NaCl, 1% NP-40, 1% Na deoxycholate and 0.1% SDS) supplemented with a complete protease inhibitor cocktail (Roche Diagnostics, Indianapolis IN), homogenized with a pellet pestle motor (Kimble/Kontes, Vineland, NJ) and subsequently centrifuged at 13,000 x g for 15 min at 4°C. For Western blots either aliquots of culture media containing the proteins secreted by the cultured cells or aliquots of the cell lysates containing cellular proteins were resolved by SDS-polyacrylamide gel electrophoresis and transferred to nitrocellulose membranes (Invitrogen, Carlsbad, CA). Blots were blocked for 1 h in Tris-buffered saline(TBS)-Tween (10 mmol/L Tris-HCl, pH 8.0, 150 mmol/L NaCl, 0.1% Tween 20) containing 5% nonfat dry milk (BioRad, Hercules, CA). The membranes were then incubated overnight at 4°C with mouse monoclonal α-SMA antibody (Abcam, 1:200), and GAPDH polyclonal rabbit antibody (Abcam, 1:2000) in a 5% nonfat dry milk/TBS-Tween solution. Membranes were then washed with TBS-Tween, and incubated for 1 h with the appropriate horseradish peroxidase-conjugated secondary antibodies (GE Healthcare, UK) diluted 3000-fold in 5% nonfat dry milk/TBS-Tween. The signals were quantified using NIH Image J software.

### Quantitative Reverse Transcription (RT)-PCR

Murine lung microvascular endothelial cells were cultured and treated as described above in duplicate wells of 12 well gelatin-treated plastic tissue culture dishes for 72 h. Following treatment, the cells were harvested with a cell lifter, washed in cold PBS, and processed for RNA extraction (RNeasy kit; Qiagen, Valencia, CA) including a genomic DNA digestion step. Total RNA (1 μg) was reverse-transcribed using Superscript II reverse transcriptase (Invitrogen) to generate first strand cDNA and various transcript levels were quantified using SYBR Green real time PCR. The primers employed are listed in S1 Table. The differences in the number of mRNA copies in each PCR were corrected for *Gapdh* endogenous control transcript levels; levels in control experiments were set at 100 and all other values were expressed as multiples of control values.

### *In Vivo* Animal Studies and Histopathology Analysis

This study was carried out in strict accordance with the recommendations in the Guide for the Care and Use of Laboratory Animals of the National Institutes of Health. The protocol was approved by the Committee on the Ethics of Animal Experiments of Thomas Jefferson University (Protocol Number 01629). All surgery was performed under sodium pentobarbital anesthesia and all efforts were made to minimize suffering. Four week old FVB/N mice purchased from The Jackson Laboratory were anesthetized and implanted subcutaneously on the interscapular region with 28 day Alzet osmotic pumps containing either saline, or 2.5 μg TGF-β1 alone, or 5 μg ET-1 alone, or 2.5 μg TGF-β1 plus 5 μg ET-1. Two animals were used per treatment group. Mice were sacrificed three weeks post-implantation. After sacrifice, the hair was removed with a commercial depilatory and a full thickness skin sample was surgically excised from the interscapular region of the animals. The skin was stretched and pinned in histopathology cassettes and fixed in phosphate buffered formalin for 24 h. The lungs were removed and the left lung was fixed by perfusion of the left pulmonary artery with paraformaldehyde for histopathological analyses. Paraffin embedded skin and lungs were stained with hematoxylin-eosin or with Masson's trichrome according to standard procedures. Dermal thickness was measured in hematoxylin-eosin stained sections viewed under 40X microscopic examination by measuring the distance between the epidermal-dermal junction and the dermal-adipose layer junction at five randomly selected fields from one sample from each animal.

### Immunohistochemical Analysis of Skin and Lung Tissues

Paraffin-embedded sections of skin and lung from animals treated with saline, TGF-β1, ET-1, or with TGF-β1 plus ET-1 were deparaffinized and dehydrated following antigen retrieval with a citric acid buffer, as described previously [53]. Slides were first incubated with blocking IgG solution for 1 h, and then incubated overnight with anti–α-SMA (Abcam; 1:100 dilution) and anti-von Willebrand antibodies (anti-vWF Dako; 1:50 dilution). IgG binding was revealed following incubation with an F(ab′) sheep anti-rabbit Cy3 antibody and an F(ab′) sheep anti-mouse fluorescein isothiocyanate—conjugated antibody (Sigma) for 1h. Nuclei were counterstained with DAPI (Jackson ImmunoResearch). Samples were examined with a Zeiss 51 confocal laser microscope to evaluate the colocalization of immunoreactivity.

### Measurement of Collagen Content of Skin and Lung Tissues

The collagen content in the lungs and skin of the same animals sacrificed for the histopathology studies was determined employing hydroxyproline assays as previously described [55]. Skin samples were obtained from areas of skin adjacent to those used for histopathology employing a 4mm diameter tissue punch and the right lung was removed. The tissues were weighed and hydrolyzed in 6M HCl overnight at 110°C. For determination of hydroxyproline content, two separate aliquots of each sample were assayed in duplicate as described previously [55].

### Statistical Analysis

Values are the mean and standard deviation of separate experiments each performed in triplicate except for the hydroxyproline assays that were performed in two separate samples and each sample was assayed in duplicate. The statistical significance of all data was assessed by Student’s two-tailed t test. *P* values less than 0.05 were considered statistically significant.

## Results

### ET-1 Potentiates TGF-β1-Induced Expression of α-SMA in Murine Lung Microvascular Endothelial Cells

**Fig 1:**
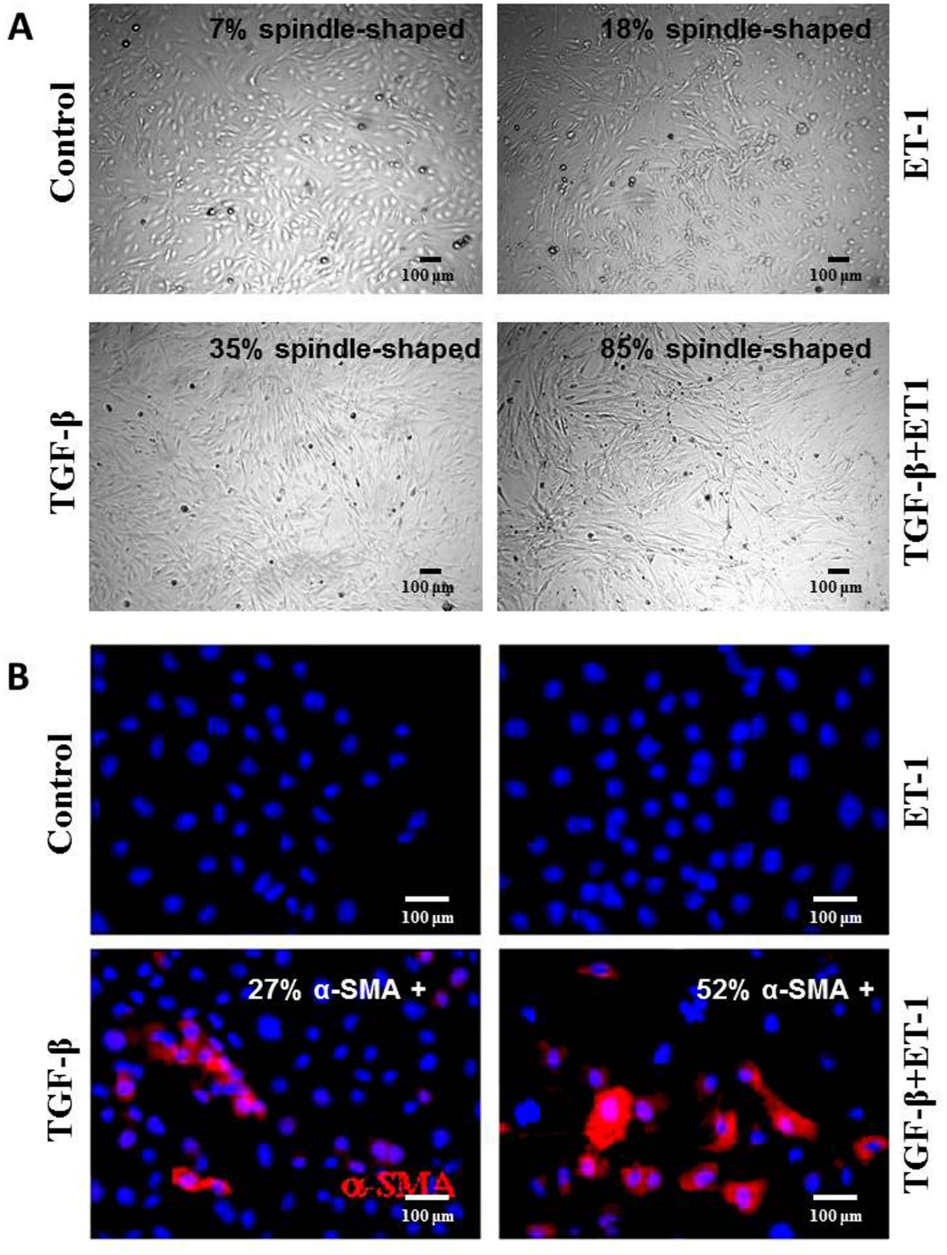
Effects of TGF-β1, or ET-1, or TGF-β1 plus ET-1 on murine lung microvascular endothelial cell morphology (A) and myofibroblast generation (B). **(A). Morphology**. Murine lung microvascular endothelial cells were cultured under control conditions (**Control**) or were treated with TGF-β1 (**TGF-β1**), ET-1 (**ET-1**) or TGF-β1 plus ET-1 (**TGF-β1+ET1**) for 72 h. Following treatment the cultures were examined by phase contrast microscope. The relative proportion of spindle-shaped cells was determined using NIH ImageJ software. Magnification: 20X. **(B)**. **Myofibroblast generation**. Murine lung microvascular endothelial cells were grown and treated on chamber slides for 72 h and then fixed and immunostained with antibodies to α-SMA. Cells were cultured under control conditions (**Control**) or were treated with TGF-β1 alone (**TGF-β1**), ET-1 alone (**ET-1**) or a combination of TGF-β1 plus ET-1 (**T+E**). Note that ET-1 alone did not induce significant changes in α-SMA expression but it markedly increased α-SMA expression induced by TGF-β1. Magnification: 40X.

We examined here the possibility that ET-1 may participate in the fibrotic process through potentiation of TGF-β1-induced EndoMT in immunopurified murine lung microvascular endothelial cells. The procedure for the isolation and culture of lung microvascular endothelial cells yielded a highly homogenous population of cells with typical morphologic characteristics of endothelial cells in culture. To assess the induction of EndoMT, we evaluated endothelial cell morphology and the expression of α-SMA, a marker of activated myofibroblasts. Saline-treated endothelial cells predominantly displayed the characteristic polygonal cobblestone morphology (Control in Fig 1A, top left), whereas treatment with TGF-β1 alone induced a transition to a more spindle-shaped fibroblast-like morphology (TGF-β1 in Fig 1A, bottom left) with 35 +/- 8% of the cells displaying this spindle-shaped morphology. ET-1 alone produced a lesser effect on endothelial cell morphology, inducing only 18 +/- 3% of the cells to assume the fibroblast-like spindle shape (ET-1 in Fig 1A, top right). In contrast, ET-1 in combination with TGF-β1 resulted in a nearly complete loss of the cobblestone morphology and the majority (85 +/-10%) of the cells acquired a spindle-shaped morphology (TGF-β1 plusET1 in Fig 1A, bottom right). The changes in cellular morphology correlated with a change in the expression of α-SMA. Saline-treated cells did not exhibit detectable expression of α-SMA (Control in Fig 1B, top left), whereas TGF-β1 treatment alone induced potent EndoMT conversion as evidenced by the appearance of numerous cells displaying strong α-SMA staining (TGF-β1 in Fig 1B, bottom left). The data also revealed that ET-1 alone had no effect on α-SMA expression (ET-1 in Fig 1B, top right), however, it displayed a potentiating effect on TGF-β1-induced α-SMA expression as evidenced by a greater intensity of fluorescence in cultures treated with ET-1 plus TGF-β1 (TGF-β1 plusET1 in Fig 1A, bottom right) in comparison with cultures treated with TGF-β1 alone. These observations were confirmed by a quantitative assessment of the proportion of cells expressing α-SMA in cultures exposed to TGF-β1 alone compared to cultures treated with TGF-β1 plus ET-1. In the cultures treated with TGF-β1 alone about 27% of cells expressed α-SMA compared with 52% in the cultures treated with both TGF-β1 plus ET-1 (compare TGF-β1 with T+E panels in Fig 1B). In all *in vitro* experiments, there were no significant effects of TGF-β1 or ET-1 alone or in combination on cellular proliferation and no cytotoxicity was detected using the WST-1 assay (data not shown).

**Fig 2:**
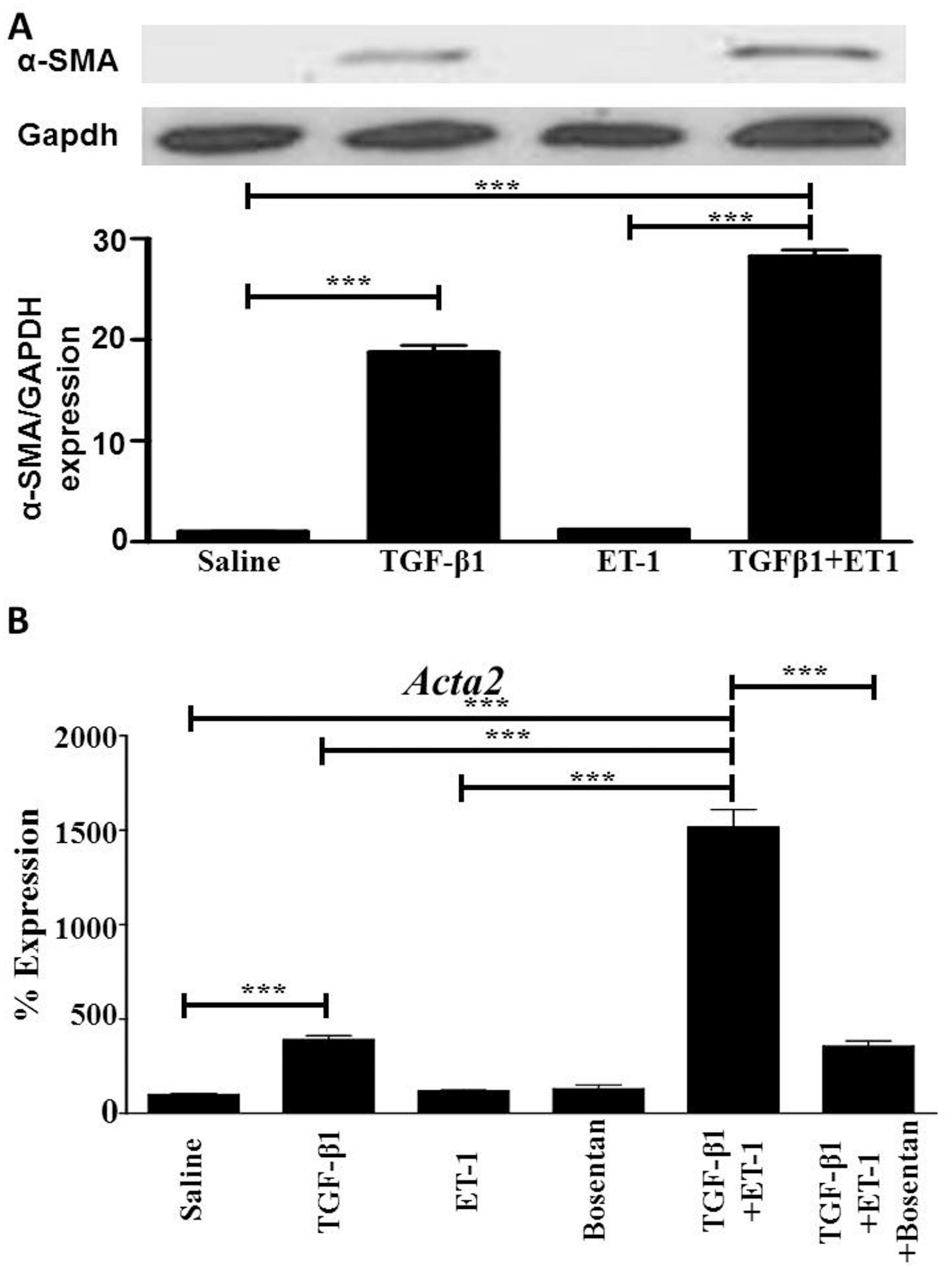
Effects of TGF-β1, or ET-1, or TGF-β1 plus ET-1 on α-SMA protein levels and *Acta2* gene expression in cultured murine lung microvascular endothelial cells. (**A). α-SMA protein levels.** The upper panel shows a Western blot of cell lysates from the same samples shown in Fig 1B probed with α-SMA (upper bands). GAPDH was used as loading control (lower bands). The Bottom panel shows a quantitative densitometry of α-SMA analyzed using NIH Image J software. (**B). *Acta2* expression.** Expression levels of *Acta2* determined by semiquantitative RT-PCR. Values represent the mean (+/- standard deviation) expression levels of three replicates of three separate experiments. C(t) values were normalized with *Gapdh*. The saline control levels were arbitrarily set at 100% expression. Values for other samples are expressed relative to the saline control. Statistical significance was determined by Student’s two-tailed t test. ***: p<0.001.

Quantitation of α-SMA gene expression and protein levels present in lung microvascular endothelial cell lysates analysed by RT-PCR and Western blot are shown in Fig 2. The Western blot results demonstrated that untreated endothelial cells did not contain detectable α-SMA levels and treatment with TGF-β1 caused an approximate 20-fold increase in α-SMA protein levels (Fig 2A). Treatment of the cells with ET-1 alone failed to induce any detectable levels of α-SMA, however, the simultaneous treatment of the cells with ET-1 and TGF-β1 resulted in a nearly 30-fold increase in α-SMA protein levels compared to either the untreated endothelial cells or to endothelial cells exposed to ET-1 alone (Fig 2A). Analysis of changes in expression levels of *Acta2*, the gene encoding α-SMA, by semi-quantitative RT-PCR revealed a similar effect of ET-1 on TGF-β1-mediated EndoMT, with TGF-β1 inducing a nearly 5-fold increase in *Acta2* expression whereas ET-1 alone did not have a significant effect (Fig 2B). In contrast, ET-1 enhanced TGF-β1-mediated *Acta2* expression to 15-fold compared to saline treated endothelial cells. This increase was abolished by exposure of TGF-β1 plus ET-1-treated lung microvascular endothelial cells to Bosentan, an inhibitor of ET-1 receptors A and B, indicating that ET-1 was indeed responsible for the increase in *Acta2* expression in samples treated with TGF-β1 plus ET-1 compared to the levels observed in samples treated with TGF-β1 alone.

### ET-1 Enhances the Expression of Types I and III Collagen during TGF-β1-Induced EndoMT

**Fig 3:**
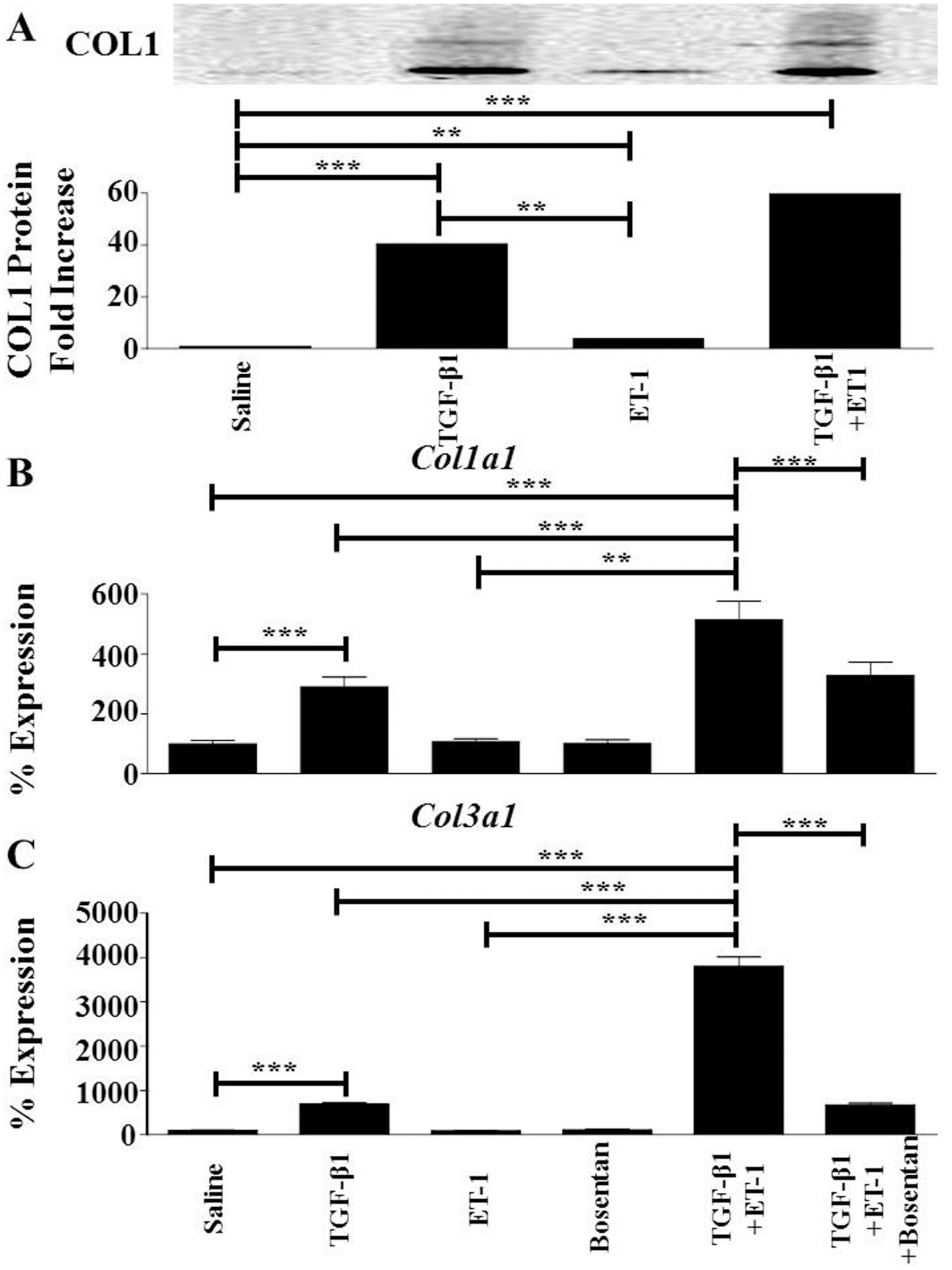
Effects of TGF-β1, or ET-1, or TGF-β1 plus ET-1 on the expression of fibrillar type I and type III collagens in murine lung microvascular endothelial cells. Murine lung endothelial cells were treated with either TGF-β1, or ET-1, or Bosentan, or with TGF-β1 plus ET-1, or with TGF-β1 plus ET-1 plus Bosentan for 72 h. (**A**). The upper panel shows a Western blot of culture supernatants probed with a Col1 primary antibody. The bottom panel shows a quantitative densitometry of bands corresponding to COL1 analyzed using NIH Image J software. (**B,C**). Expression levels of *Col1a1* (**B**), and *Col3a1* (**C**) as determined by semiquantitative RT-PCR. Values represent the mean (+/- standard deviation) expression levels of three replicates of three separate experiments. C(t) values were normalized with *Gapdh*. The saline control levels were arbitrarily set at 100% expression. Values for other samples are expressed relative to the saline control. Statistical significance was determined by Student’s two-tailed t test. **: p < 0.01; ***: p<0.001. T: TGF-β1; E: ET-1; B: Bosentan.

Another effect of TGF-β-mediated EndoMT is an increase in the expression of the mesenchymal-specific fibrillar type I and type III collagens (*Col1a1* and *Col3a1*, respectively). Western blot assessment showed that TGF-β1 induced a nearly 30-fold increase in levels of type I collagen (COL1) present in the culture supernatants compared to saline-treated cells and that ET-1 treatment alone induced a minor but significant 2.5 fold increase in the levels of COL1 in these cells (Fig 3A). In contrast, TGF-β1 in combination with ET-1 induced a nearly 45-fold increase in COL1 levels. Fig 3B and 3C show the effects of TGF-β1 alone, ET-1 alone, or ET-1 plus TGF-β1 on *Col1a1* and *Col3a1* gene expression. TGF-β1 alone induced a 3-fold increase in *Col1a1* expression (Fig 3B) and a 7-fold increase in *Col3a1* expression (Fig 3C) whereas ET-1 alone did not significantly alter the expression levels of either gene compared to those measured in saline-treated endothelial cells. ET-1 in combination with TGF-β1, however, increased the levels of *Col1a1* and *Col3a1* expression to 5-fold and ~40-fold, respectively, compared to levels in saline treated cells. Bosentan abrogated the stimulation of ET-1 on the TGF-β1-effects on *Col1a1* and *Col3a1* expression.

### ET-1 Potentiates TGF-β1-Induced Increased Expression of Mesenchymal Cell-Specific Transcription Factors

**Fig 4:**
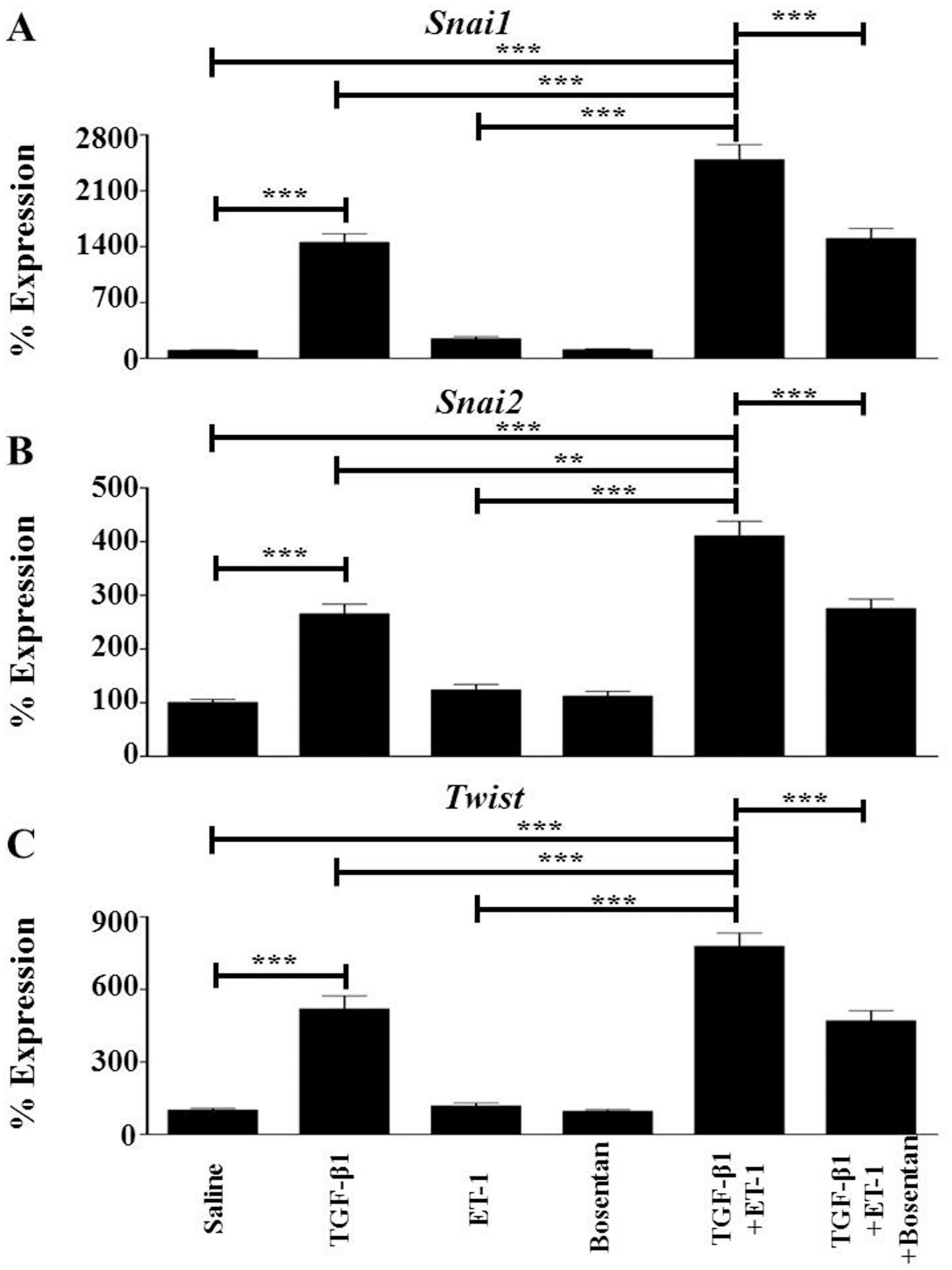
Effects of TGF-β1, or ET-1, or TGF-β1 plus ET-1 on the expression of mesenchymal cell-specific transcription factors in murine lung microvascular endothelial cells. Murine lung microvascular endothelial cells were treated with either TGF-β1, or ET-1, or bosentan, or with TGF-β1 plus ET-1, or with TGF-β1 plus ET-1 plus Bosentan for 72 h. Expression levels of *Snai1*(**A**), *Snai2* (**B**) and *Twist1* (**C**) were determined by semiquantitative RT-PCR. Values represent the mean (+/- standard deviation) expression levels of three replicates of three separate experiments. C(t) values were normalized with *Gapdh*. The saline control levels were arbitrarily set at 100% expression. Values for other samples are expressed relative to the saline control. Statistical significance was determined by Student’s two-tailed t test. **: p < 0.01; ***: p<0.001.

It was previously shown that TGF-β induced a marked increase in the expression of transcription factors and transcription factor coactivators involved in EndoMT [33–35]. The results obtained here confirmed these studies as it was found that the levels of *Snai1* in TGF-β1-treated cells were ~15-fold greater than those measured in untreated endothelial cells (Fig 4A), the levels of *Snai2* were ~2.5-fold greater (Fig 4B), and the levels of *Twist1* were ~5-fold greater (Fig 4C). Treatment with ET-1 alone did not significantly affect expression of any these genes, whereas ET-1 potentiated TGF-β1-mediated increases in expression of *Snai1* (to ~25-fold), *Snai2* (to ~4-fold) and *Twist1* (to ~8-fold). Consistent with these observations, pretreatment of endothelial cells with Bosentan abrogated the ET-1-mediated increases in *Snai1*, *Snai2* and *Twist1* expression, returning their expression levels to those observed with TGF-β1 treatment alone.

### ET-1 Enhances Expression of TGF-β Genes during TGF-β1-Mediated EndoMT

**Fig 5:**
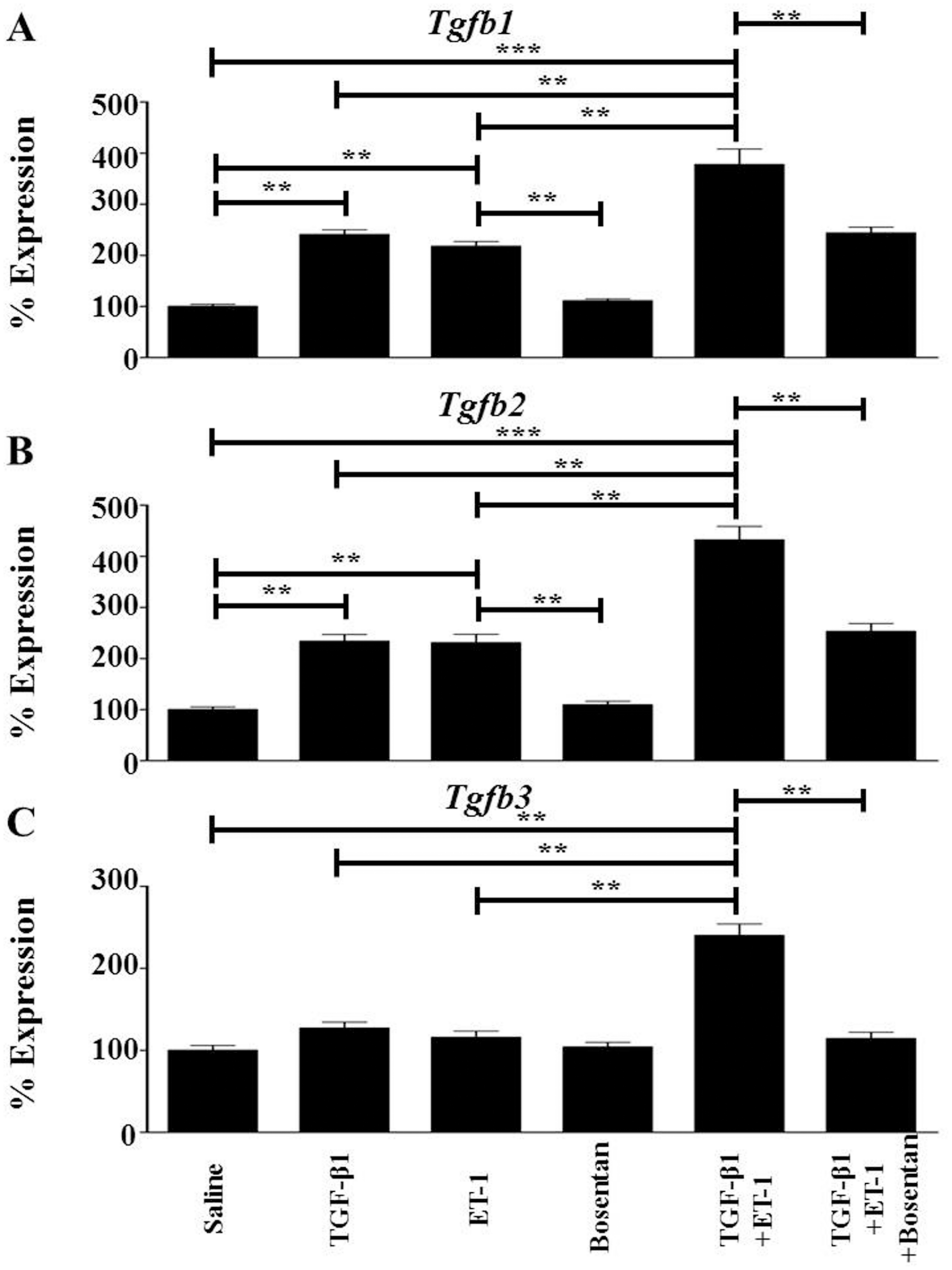
Effects of TGF-β1, or ET-1, or TGF-β1 plus ET-1 on the expression of TGF-β isoforms in murine lung microvascular endothelial cells. Murine lung microvascular endothelial cells were treated with either TGF-β1, or ET-1, or Bosentan, or with TGF-β1 plus ET-1, or TGF-β1 plus ET-1 plus Bosentan for 72 h. Expression levels of *Tgfb1*(**A**), *Tgfb2* (**B**) and *Tgfb3* (**C**) were determined by semiquantitative RT-PCR. Values represent the mean (+/- standard deviation) expression levels of three replicates of three separate experiments. C(t) values were normalized with *Gapdh*. The saline control levels were arbitrarily set at 100% expression. Values for other samples are expressed relative to the saline control. Statistical significance was determined by Student’s two-tailed t test. **: p < 0.01; ***: p<0.001.

To gain insight into the mechanisms responsible for the potentiating effects of ET-1 on TGF-β1-induced EndoMT, we examined the effect of ET-1 on the expression levels of the TGF-β isoforms, *Tgfb1*, *Tgfb2* and *Tgfb3*. Either TGF-β1 or ET-1 significantly increased expression of *Tgfb1* (Fig 5A) and of *Tgfb2* (Fig 5B). However, ET-1 potentiated TGF-β1-mediated increases in *Tgfb1* and *Tgfb2*, inducing a 4-fold increase for either of these genes compared to untreated cells. Although neither TGF-β1 alone nor ET-1 alone caused a significant change in the expression of *Tgfb3*, the combination of TGF-β1 plus ET-1 significantly increased expression of *Tgfb3* by 2-fold (Fig 5C). Bosentan abrogated ET-1 plus TGF-β1-mediated increases in expression of all TGF-β isoforms.

### ET-1 Induces Expression of TGF-β Receptor Genes during TGF-β-Mediated EndoMT

**Fig 6:**
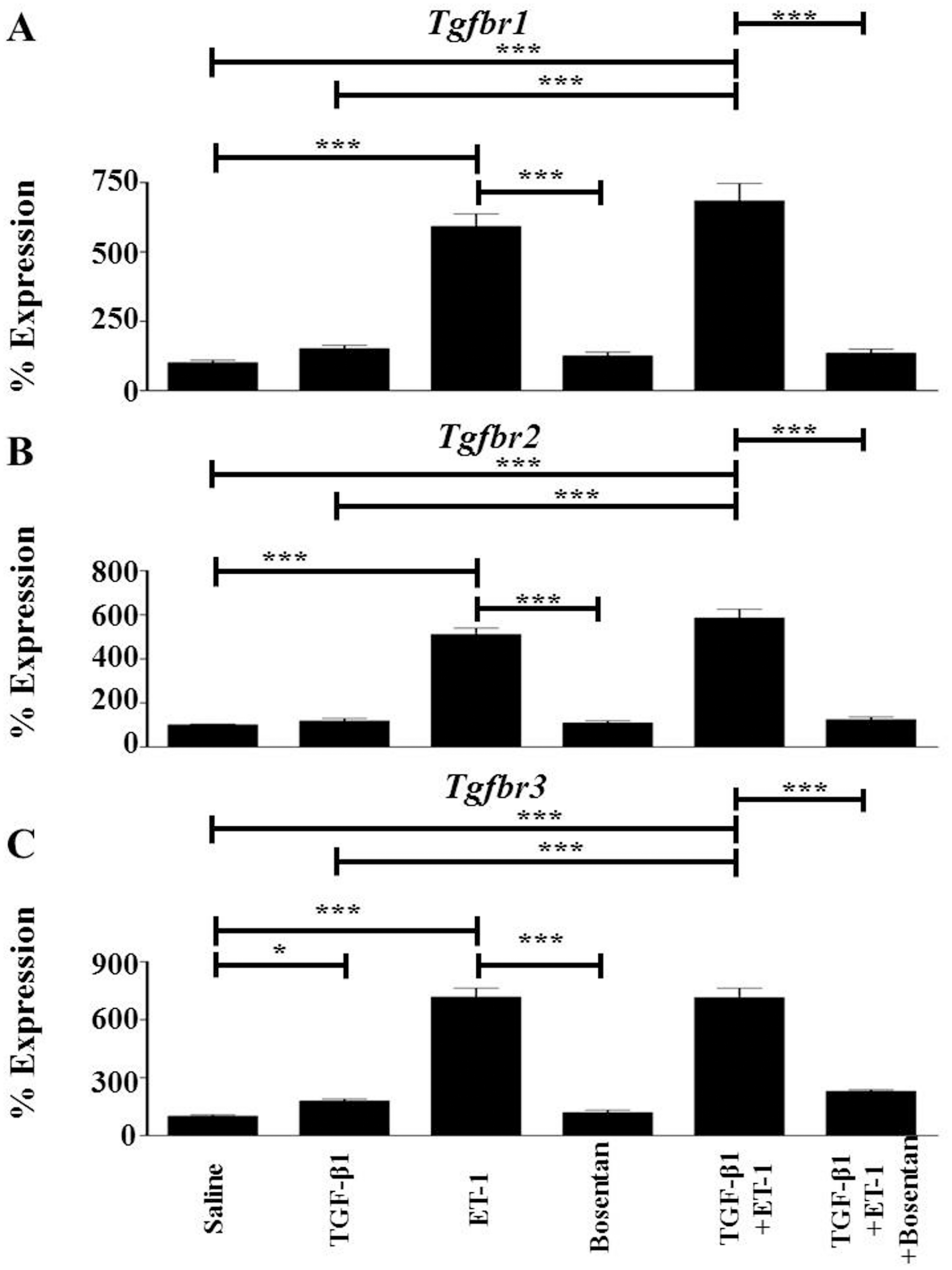
Effects of TGF-β1, or ET-1, or TGF-β1 plus ET-1 on the expression of TGF-β receptors in murine lung microvascular endothelial cells. Murine lung microvascular endothelial cells were treated with either TGF-β1, or ET-1, or Bosentan, or with TGF-β1 plus ET-1, or with TGF-β1 plus ET-1 plus Bosentan for 72 h. Expression levels of *Tgfbr1*(**A**), *Tgfbr2* (**B**) and *Tgfbr3* (**C**) were determined by semiquantitative RT-PCR. Values represent the mean (+/- standard deviation) expression levels of three replicates of three separate experiments. C(t) values were normalized with *Gapdh*. The saline control levels were arbitrarily set at 100% expression. Values for other samples are expressed relative to the saline control. Statistical significance was determined by Student’s two-tailed t test.*: p<0.05; **: p < 0.01; ***: p<0.001.

In these studies, we assessed the effects of TGF- β1, ET-1, or TGF-β1 plus ET-1 on the expression levels of three TGF-β receptors (Fig 6). Treatment with TGF-β1 alone had no significant effect on the expression of *Tgfbr1* and *Tgfbr2* (Fig 6A and 6B) but induced a 2-fold increase in *Tgfbr3* (Fig 6C), whereas ET-1 alone significantly induced increased expression of all three genes (6-fold for *Tgfbr*, 5-fold for *Tgfbr2* and 7-fold for *Tgfbr3*) compared to saline treated controls. However, in contrast with the other experiments reported here, no additional stimulatory effects were observed when endothelial cells were exposed to the combination of TGF-β1 and ET-1. Exposure to Bosentan suppressed all ET-1-mediated increases in TGF-β receptor genes.

### ET-1 Stimulates TGF-β1-Induced Skin and Lung Fibrosis *In Vivo*

**Fig 7:**
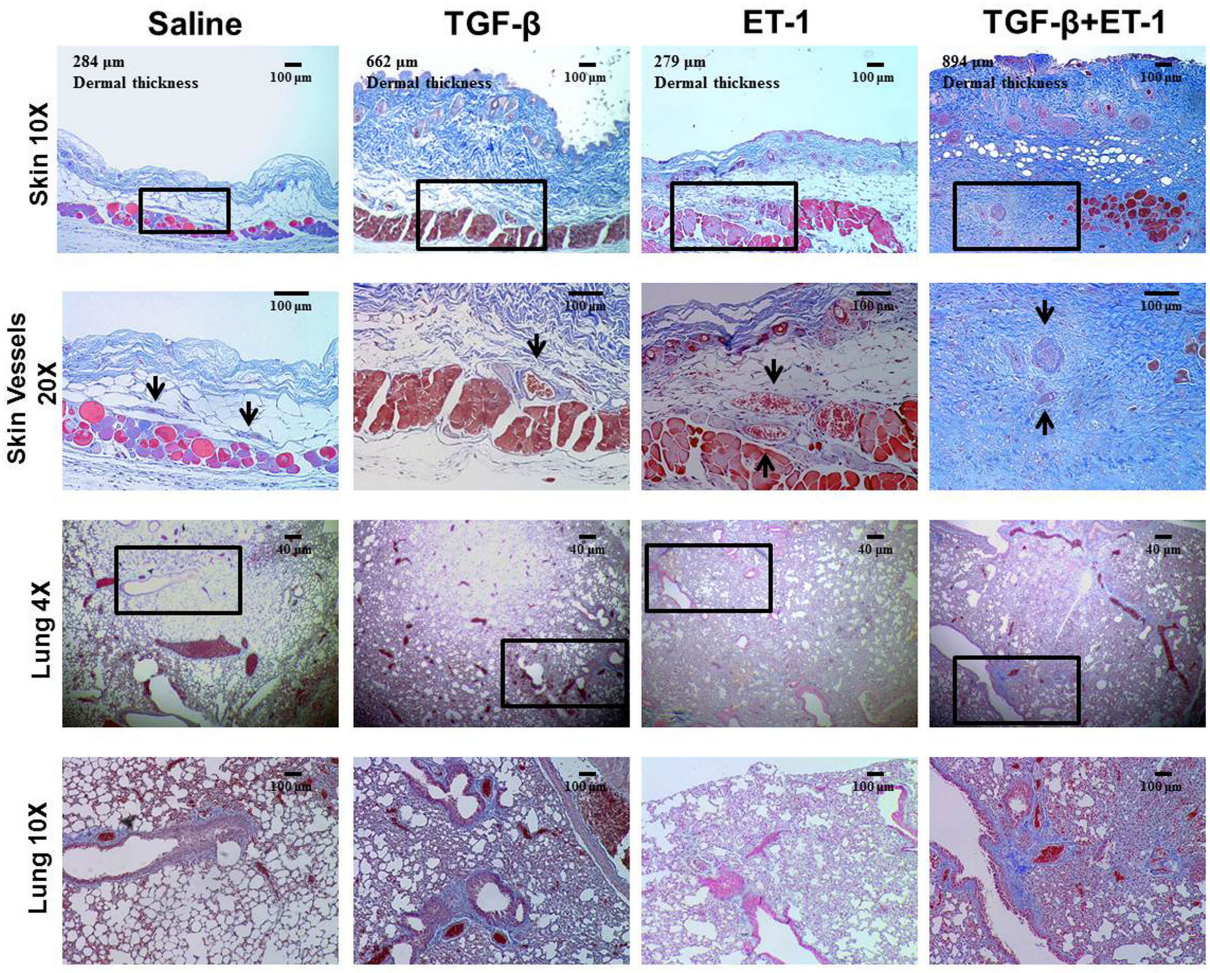
Histopathology of skin and lung isolated from mice treated with either saline, or TGF-β1, or ET-1 or TGF-β1plus ET-1. Trichrome stained sections of skin and lungs from mice implanted subcutaneously with osmotic pumps delivering either saline (left column), or 2.5 μg TGF-β1 (middle column), or 5 μg ET-1, or 2.5 μg TGF-β1+5 μg ET-1 during a 28 day period are shown. Skin samples (upper row) Magnification: 20X. Black boxes in the low power (10X images in the top row are shown at 20X magnification to demonstrate changes in blood vessel (black arrows) morphology). Black boxes in the low power (4X) images of lungs in the third row are displayed at higher magnification (10X) in the bottom row. Note the replacement of alveolar structure with areas of fibrotic tissue consolidation in the lungs and the marked increase in collagen deposition in the skin and lungs from TGF-β1-treated mice and noticeable greater increase in samples from mice receiving both TGF-β1 plus ET-1. The samples from mice receiving only ET-1 show only a minimal increase in tissue collagen accumulation.

These studies were performed to validate *in vivo* the results obtained in the *in vitro* studies indicating that ET-1 stimulated the pro-fibrotic effects of TGF-β. Four week old FVB/N mice were implanted subcutaneously with osmotic pumps containing either saline, or TGF-β1, or ET-1, or TGF-β1 plus ET-1. Mice were sacrificed 3 weeks post-implantation and a sample of skin was removed. Both lungs were also isolated. Trichrome staining of these tissues is displayed in Fig 7. The skin samples from mice implanted with pumps containing TGF-β1 revealed a nearly 2 fold increase in dermal thickness compared to the saline-treated animals (Fig 7, upper row). The TGF-β1-treated skin also displayed mild to moderate multifocal to coalescing fibrosis of the dermis and of the panniculus carnosum muscle. Increased collagen accumulation was also demonstrated in the deeper dermis as well as surrounding numerous blood vessels in the skin (Fig 7, second row). Mice treated with ET-1 alone exhibited minimal changes in dermal thickness (Fig 7, upper row) and in the deep dermal histology (Fig 7, second row). In contrast, in mice treated with TGF-β1 plus ET-1, the skin showed extensive dermal and hypodermal accumulation of dense connective tissue with marked fibroplasia (Fig 7, top and middle rows), and a 3.1 fold increase in dermal thickness.

The histopathology of the lungs is shown at low magnification in Fig 7, third row with higher 10X magnification in Fig 7, bottom row. The lungs of the TGF-β1-treated mice displayed perivascular, peribronchiolar and interstitial fibrosis that was not evident in saline-treated animals as well as numerous areas of fibrotic consolidation whereas the lungs from the ET-1-treated animals showed only a minimal increase in trichrome staining compared to lungs isolated from the saline-treated control animals (Fig 7, bottom row). The lungs of mice treated with TGF-β1 plus ET-1 displayed marked perivascular, peribronchiolar and severe interstitial fibrosis with large areas of parenchymal replacement by fibrotic tissue (Fig 7, third and bottom rows) compared to the untreated control and to ET-1 alone-treated mice.

### Coexpression of α-SMA and vWF in Perivascular Cells in the Lungs of Mice Treated with Either Saline, or TGF-β1, or ET-1 or TGF-β1 Plus ET-1

**Fig 8:**
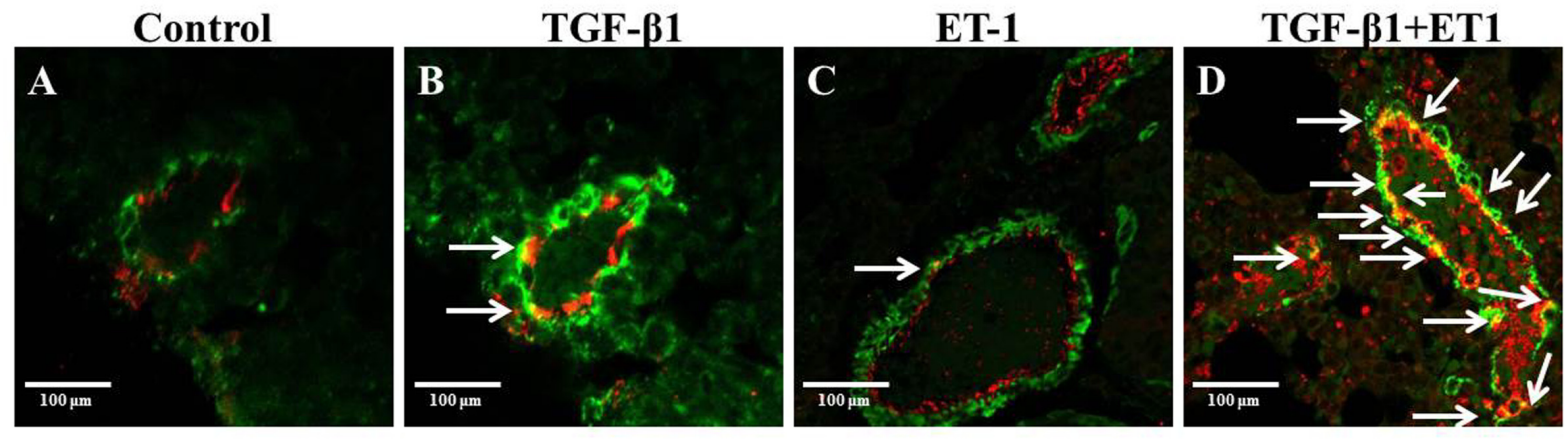
Coexpression of vWF and α-SMA in small vessels of the lungs from mice treated with either saline, or TGF-β1, or ET-1 or TGF-β1 plus ET-1. Confocal microscopy staining for vWF (red) and α-SMA (green) in the lungs from mice treated with saline (**A**), TGF-β1 (**B**), ET-1 (**C**), or TGF-β1 + ET-1 (**D**). DAPI was used for counterstaining of nuclei. Endothelial cells expressing vWF (red) are seen lining the large and small vessels of the lung. Activated myofibroblasts expressing α-SMA (green) are seen surrounding the vessels and in the interstitium. Cells co-staining for vWF and α-SMA (yellow; white arrows) in the small vessels represent cells in the process of endothelial-to-mesenchymal transition are observed in the TGF-β1 (**B**), ET-1 (**C**), or TGF-β1 + ET-1 (**D**)-treated animals. No double positive cells were observed in lungs from mice injected with saline (**A**). Magnification: 40X.

Immunofluorescence staining of paraffin-embedded sections of lungs from saline-treated control mice for the presence of α-SMA and vWF show clearly delineated expression of each of these proteins with no detectable overlap of expression (Fig 8A). However, in mice treated with TGF-β1 alone, the number of α-SMA-positive cells increased, particularly surrounding the small vessels in the lung (Fig 8B). Interestingly, a small percentage of vWF positive cells simultaneously displayed the expression of α-SMA surrounding these vessels suggesting that a certain proportion of these cells could be undergoing endothelial-to-mesenchymal transition (white arrows). In lungs from animals treated with ET-1 alone, an increased number of α-SMA-positive cells was observed compared to control animals, however, this increase was less than that observed in animals treated with TGF-β1 alone (Fig 8C). In the animals treated with both TGF-β1 plus ET-1, a massive increase in cells positive for α-SMA as well as for cells co-staining for vWF and α-SMA (white arrows) was observed (Fig 8A).

## Discussion

Activated myofibroblasts are considered the crucial effector cells in the development of pathologic tissue fibrosis. Given their important role during normal tissue repair reactions as well as in pathologic fibrogenesis there has been intense investigation aimed at the identification if their origins [3,4]. Recently, EndoMT has been recognized as an important source for the generation of activated myofibroblasts in various animal models of tissue fibrosis and in several human fibrotic diseases [13–15]. EndoMT is a complex biological process leading to the transition of an endothelial cell phenotype into the phenotype of mesenchymal cells with the initiation of expression of α-SMA and the upregulated expression of fibrotic tissue components including the fibrillar type I and type III collagens. TGF-β plays a crucial role in the induction and regulation of EndoMT, both during embryonic development [12], as well as in animal models of organ-specific fibrosis, and in various human fibrotic diseases [16–26]. Although numerous studies have described important pro-fibrotic effects of ET-1 [40–46], the possibility that ET-1 may contribute to EndoMT has not been extensively studied.

The results described here show that ET-1 enhanced the TGF-β1-mediated EndoMT process resulting in potent stimulation of the expression of multiple mesenchymal cell-specific genes including *Acta2*/α-SMA (Fig 2), the interstitial collagens *Col1a1* and *Col3a1* (Fig 3B and 3C), and relevant transcriptional coactivators such as *Snai1* and *Twist1* (Fig 4) in murine lung microvascular endothelial cells. The Snail and Twist transcription factors are well characterized regulators of mesenchymal identity and also play a role in EndoMT during embryonic development as well as in pathologic fibrotic conditions like cancer [56,57] Most intriguingly, the results also demonstrate that ET-1 was able to induce a potent increase in the expression of TGF-β1 and TGF-β2 (Fig 5) and of the three TGF-β receptors (Fig 6). It is plausible that the ET-1 mediated upregulation of TGF-β receptors may result in increased sensitivity of these cells to the TGF-β present in the microenvironment. Furthermore, ET-1 may be capable of initiating an autocrine loop by inducing expression of the TGF-β ligands resulting in amplification of the induction of EndoMT. It has previously been reported that in fibroblasts co-cultured with keratinocytes, mechanical stress induces the expression of ET-1 in these fibroblasts which can then augment the effects of TGF-β in triggering their transdifferentiaton into activated myofibroblasts with upregulated expression of α-SMA [58] and that the formation of stress fibers was an early event in this process that was required for myofibroblast transdifferentiation. We did not observe the formation of stress fibers during our experiment despite the occurrence of EndoMT, however, it is possible that the sequence and kinetics of the events involved in the transdifferentiation of quiescent fibroblasts to activated myofibroblasts may differ from those required for the transition of endothelial lineage cells to activated myofibroblasts (Fig 8).

**Fig 9:**
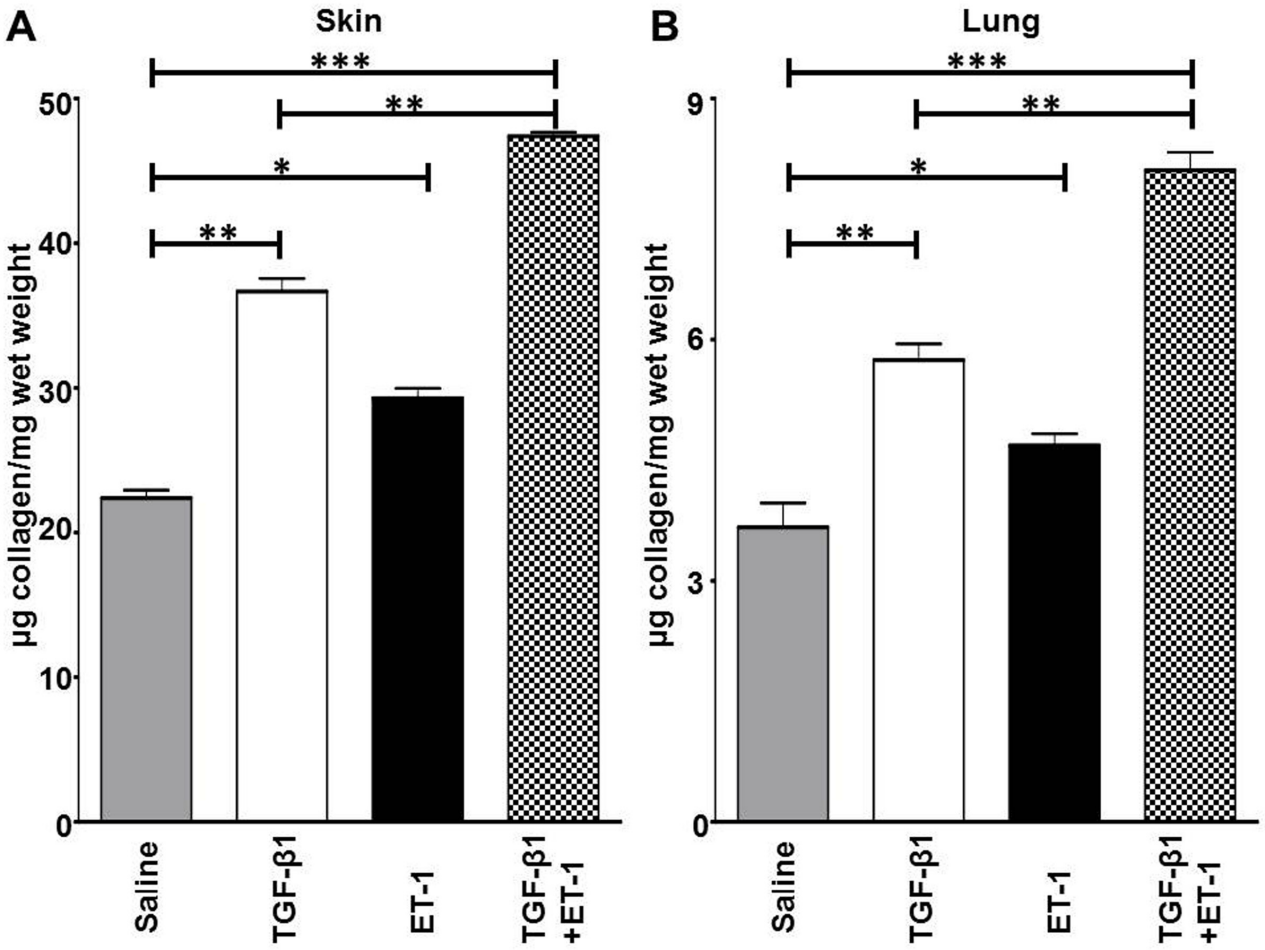
Collagen content of skin and lung isolated from mice treated with either saline, or TGF-β1, or ET-1, or TGF-β1 plus ET-1. Hydroxyproline assay of collagen content of skin isolated from the delivery site of the subcutaneous pumps (**A**) and of the lungs (**B**) are displayed. One sample of skin and one sample of lung tissue from each experimental condition were hydrolyzed and aliquots of the hydrolysates were assayed for their hydroxyproline content. Results are shown as the mean μg of collagen /mg of wet weight of three aliquots from the same hydrolyzed tissue sample. Statistical significance was determined by Student’s two-tailed t test.*: p<0.05; **: p < 0.01; ***: p<0.001.

These *in vitro* observations were confirmed by *in vivo* studies in mice that had received continuous administration of either TGF-β1 alone, ET-1 alone, or the combination of TGF-β1 plus ET-1 as shown in Figs 7, 8 and 9. In mice treated with TGF-β1 alone, an increase in dermal thickness with fibrotic tissue accumulation in the dermis and surrounding the subdermal vessels was observed. An accumulation of interstitial, peribronchial and perivascular collagen in the lungs was also noted in response to TGF-β1. Only a minor increase in dermal or lung collagen was observed in mice treated with ET-1 alone. However, the fibrotic changes in skin and lung tissue histology observed in mice treated with TGF-β1 were accentuated in tissues from mice that had been treated with the combination of TGF-β1 plus ET-1 (Fig 7). Costaining the lungs with antibodies for the endothelial lineage marker vWF and for the marker of activated myofibroblasts, α-SMA indicated that a small number of cells undergo EndoMT in mice treated with either TGF-β1 or ET-1 alone but that treatment with TGF-β1 plus ET-1 greatly increases the number of cells undergoing EndoMT to produce activated myofibroblasts.

A quantitative assessment of the collagen content demonstrated significant increases in both skin and lung tissues from mice receiving TGF-β1 whereas less but still significant increases in tissue morphology or collagen content were observed in the skin and lungs of mice treated with ET-1 alone (Fig 8). However, the combined treatment with TGF-β1 plus ET-1 resulted in a significantly greater increase in collagen content compared to that of tissues from animals receiving only TGF-β1.

**Fig 10:**
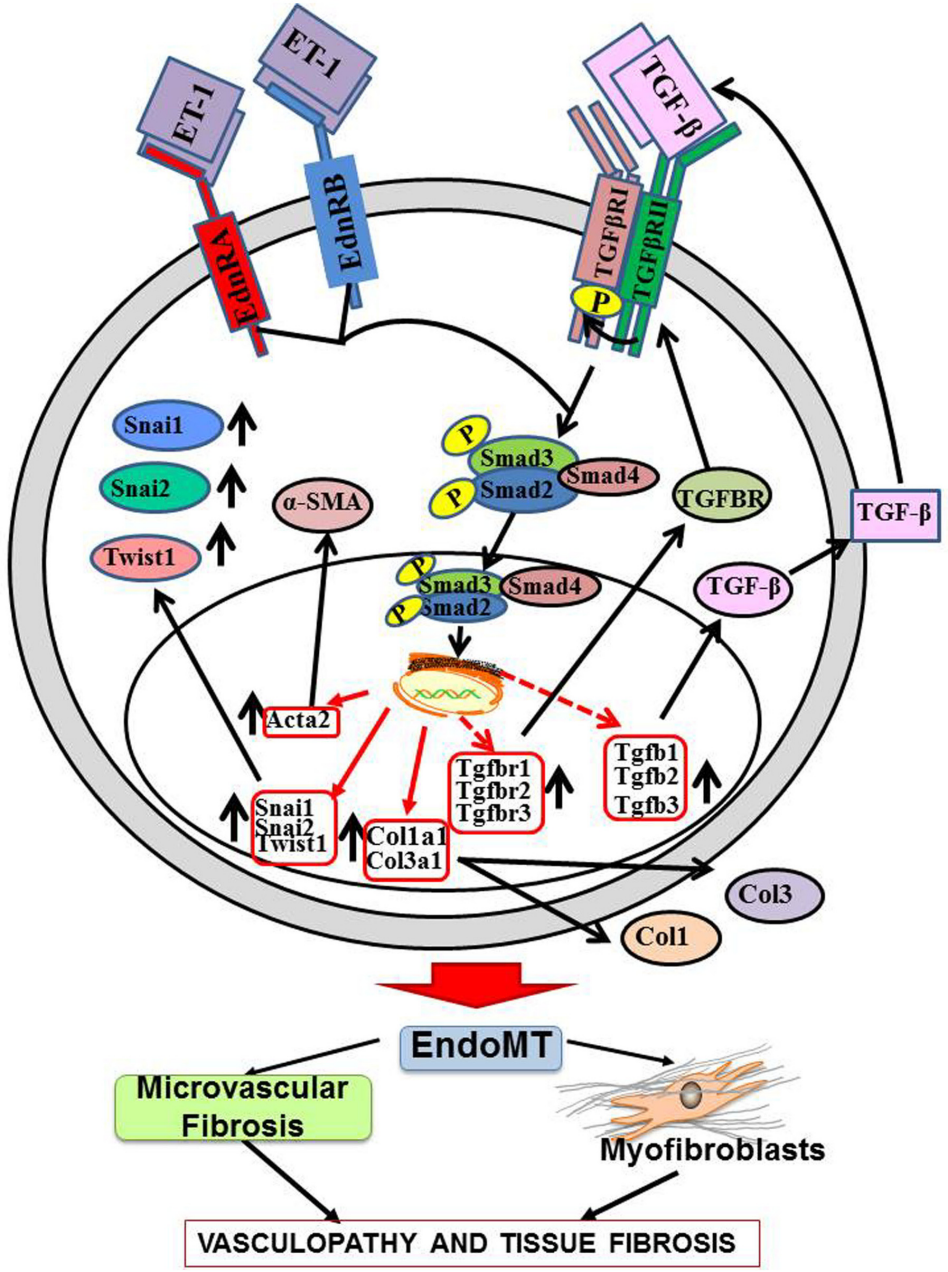
Diagrammatic representation of a proposed autocrine pathway of the stimulation of TGF-β-mediated EndoMT by ET-1. Following TGF-β dimerization and dimer binding to its cognate cell surface receptor there is activation of the TGF-β receptor 2 which in turn leads to phosphorylation of the TGF-β receptor 1. The phosphorylated TGF-β1 induces the phosphorylation of Smad2/3, the recruitment of Smad4, and the translocation of the complex to the nucleus. Binding of Smads to specific gene promoter elements results in the induction of increased transcription of TGF-β1 target genes (indicated by solid red arrows). When exposed to TGF-β alone, endothelial cells undergo EndoMT, resulting in increased expression and production of *Acta2*/α-SMA mediated by the increased levels of mesenchymal-specific transcription factors Snail1, Snail2 and Twist1. The phenotypic transition of endothelial cells to activated myofibroblasts is responsible for vascular and tissue fibrosis. Exposure of endothelial cells to ET-1 plus TGF-β synergistically induces increased expression of TGF-β and its receptors (indicated by red broken arrows) with the increased production of the corresponding proteins generating an autocrine loop that maintains and amplifies the EndoMT-inducing effects of TGF-β resulting in progressive tissue fibrosis.

The results described here indicate that ET-1 potentiates the TGF-β1-mediated acquisition of a mesenchymal cell phenotype by lung endothelial cells as well as the exaggerated tissue fibrotic process induced by TGF-β1. These observations identify a novel mechanism for ET-1 participation in the development of tissue fibrosis and proliferative vasculopathy mediated through the potentiation of TGF-β-induced EndoMT. Furthermore, the assessment of gene expression levels of TGF-β isoforms and of their corresponding receptors described here suggest that in the lung microvascular endothelial cells ET-1 produced by these cells may initiate a profibrotic autocrine pathway. In this pathway, ET-1 stimulates the expression of TGF-β1 and TGF-β2 (Fig 5A and 5B), and induces a parallel stimulation of the expression of the TGF-β receptors (Fig 6), thus, creating a potent autocrine mechanism of activation of TGF-β-induced EndoMT resulting in strong pro-fibrotic effects as illustrated diagrammatically in Fig 10. These data provide a novel mechanistic interaction between TGF-β and ET-1 and support the concept that ET-1 may play an important pathogenetic role during the earliest stages of development of fibrotic processes involving TGF-β-mediated EndoMT. These results also expand the previously described interactions between TGF-β and ET-1 during normal tissue repair reactions or in the course of organ-specific or systemic pathologic fibrogenesis [59–64].

## Supporting Information

S1 Table Primers employed for quantitative real time PCR. (DOC)
