# Supplementary material for: Stimulation of Transforming Growth Factor-β1-Induced Endothelial-To-Mesenchymal Transition and Tissue Fibrosis by Endothelin-1 (ET-1): A Novel Profibrotic Effect of ET-1
Source: PLoS One. 2016 Sep 1;11(9):e0161988. doi: 10.1371/journal.pone.0161988 (PMC5008786; doi:10.1371/journal.pone.0161988)
Supplement: S1 Table — (DOC) [file pone.0161988.s001.doc]

| **S1 Table. Primers employed for quantitative real time PCR.** | | |
| --- | --- | --- |
| **Gene** | **Forward Primer (5'-3')** | **Reverse Primer (5'-3')** |
| *Acta2* | CGATAGAACACGGCATCATCA | CAGTGTCGGATGCTCTTCAG |
| *Collal* | GCATGGCCAAGAAGACATCG | TCCACGTCTCAGCATTGGG |
| *Col3al* | AGCTTTGTGCAAAGTGGAACCTGG | CAAGGTGGCTGCATCCCAATTCAT |
| *Fnl* | TCCAGGACAACAGCATCAGTGTCA | CCACAGTGGGTTGCAAACCTTCAA |
| *Gapdh* | CATGGCCTTCCGTGTTCCTA | CCTGCTTCACCACCTTCTTGAT |
| *Snail* | TTGTGTCTGCACGACCTGTGGAAA | TCTTCACATCCGAGTGGGTTTGCA |
| *Snai2* | ACTACAGCGAACTGGACACACACA | AAAGGCCACTGGGTAAAGGAGAGT |
| *Tgtbl* | CTGAACCAAGGAGACGGAATAC | GGGCTGATCCCGTTGATTT |
| *Tgtb2* | GTACTACGCCAAGGAGGTTTAT | GACGATTCTGAAGTAGGGTCTG |
| *Tgtb3* | AGGGCAGTCAGAGGAAGAA | GATCCTGCCGGAAGTCAATATAA |
| *Tgtbrl* | GGGCTTAGTGTTCTGGGAAA | CCGATGGATCAGAAGGTACAAG |
| *Tgtbr2* | AGGGCAGTCAGAGGAAGAA | GATCCTGCCGGAAGTCAATATAA |
| *Tgtbr3* | GCTCGATGGAAATGCTACCT | CCTTAGTGACAGACACCTCAAC |
| *Twist1* | ATGTCCGCGTCCCACTA | CCAGAGTCTCTAGACTGTCCAT |
